# Recent advances in understanding *Listeria monocytogenes *infection: the importance of subcellular and physiological context

**DOI:** 10.12688/f1000research.11363.1

**Published:** 2017-07-13

**Authors:** Daryl J. V. David, Pascale Cossart

**Affiliations:** 1 Unité des Interactions Bactéries-Cellules, Department of Cell Biology and Infection, Institut Pasteur, Paris, France

**Keywords:** Listeria monocytogene, listeriosis, Foodborne disease

## Abstract

The bacterial pathogen *Listeria monocytogenes* ( *Lm*) is the causative agent of listeriosis, a rare but fatal foodborne disease. During infection, *Lm* can traverse several host barriers and enter the cytosol of a variety of cell types. Thus, consideration of the extracellular and intracellular niches of *Lm* is critical for understanding the infection process. Here, we review advances in our understanding of *Lm* infection and highlight how the interactions between the host and the pathogen are context dependent. We discuss discoveries of how *Lm* senses entry into the host cell cytosol. We present findings concerning how the nature of the various cytoskeleton components subverted by *Lm* changes depending on both the stage of infection and the subcellular context. We present discoveries of critical components required for *Lm* traversal of physiological barriers. Interactions between the host gut microbiota and *Lm* will be briefly discussed. Finally, the importance of *Lm* biodiversity and post-genomics approaches as a promising way to discover novel virulence factors will be highlighted.

## Introduction

*Listeria monocytogenes* ( *Lm*) is ubiquitous in the environment and potentially an enteropathogen. *Lm*is the causative agent of the foodborne disease listeriosis and is thus a major concern in the food industry. *Lm* switches between saprophytism and virulence depending on its environmental context. *Lm* can replicate intracellularly in a variety of cell types, can traverse several host barriers, and has long been used as a model of infection. The capacity of *Lm* to infect multiple tissues has underlined the cell-type-dependent role of different bacterial and host proteins.

*Lm* can infect a wide variety of cell types during its dissemination in the host, invading both phagocytic and non-phagocytic cells in a variety of tissues ^1^. Following internalization into the host cell, the bacterium escapes its membrane-bound vacuole and replicates within the cytosol. The bacterium then subverts the host cytoskeleton, inducing characteristic actin “comet tails” to drive both intracellular and intercellular movements. The most important virulence factor (in addition to actin assembly-inducing protein [ActA] ^2^ responsible for the actin-based motility, and the two invasion proteins internalin A [InlA] and internalin B [InlB]), is certainly listeriolysin O (LLO) ^3^. This pore-forming toxin appears to be a multifaceted factor involved in several steps of infection, before bacterial entry into cells, at the level of the escape from the vacuole, and in the cytosol.

Here, we review recent advances in the understanding of *Lm* infection with a particular focus on the importance of taking into account the subcellular and physiological environmental context. We highlight some recently discovered cues used by *Lm* to sense entry into the host as a signal to regulate virulence. Furthermore, we discuss new aspects of *Lm* subversion of the actin cytoskeleton. We also provide recent updates on how *Lm* crosses physiological barriers, notably the small intestine and placenta. Recent work has also uncovered the interaction between *Lm* and the host gut microbiota, highlighting the importance of considering not only standard laboratory strains of *Lm* but also other strains as a source of discovery of novel virulence factors.

## Subversion of host cell processes

### Detection of the host cell environment: the role of glutathione and L-glutamine

Upon entry into the host cell, *Lm* is known to modify its transcriptional program ^4– 7^. The transcription factor positive regulatory factor A (PrfA) is a master transcriptional activator of genes necessary for *Lm* pathogenesis, including *prfA* itself ^8, 9^. The expression of *prfA* is regulated by a variety of cues, allowing *Lm* to adapt to different environments. PrfA translation is known to be dependent on temperature, with higher translation levels at 37°C compared to 30°C ^10^. Recently, it was found that the scarcity of branched-chain amino acids, as would be encountered by *Lm* during infection, leads to upregulation of *prfA* transcription ^11, 12^. Furthermore, glutathione, abundant within the host cytosol, has been uncovered as an allosteric activator of PrfA protein activity ^13, 14^. In addition to activating PrfA, glutathione was discovered to covalently attach to a conserved cysteine on LLO ^15^. This S-glutathionylation abolishes LLO hemolytic activity, but the precise mechanism by which this reversible post-translational modification affects infection is unknown.

L-glutamine, abundant within host blood plasma and host cell cytosol, has recently been reported as another major cytosolic cue for the upregulation of virulence genes in *Lm* ^16^. It is currently unknown whether L-glutamine, similarly to glutathione, affects PrfA activity at the post-translational level.

To further investigate the cues sensed by *Lm* for the regulation of virulence, a screen was performed to identify *Lm* genes required for expression of the surface protein ActA ^17^. Interestingly, most of the genes important for ActA expression are implicated in bacterial redox homeostasis. Since the host cell can induce oxidative stress as a means of antibacterial activity, redox changes may serve as another cue for the regulation of virulence genes in *Lm*.

The discovery of novel environmental cues sensed by *Lm* will continue to be important for the study of the infectious process. Indeed, earlier studies have shown an intracellular upregulation of some virulence factors, e.g. InlK or LntA, but the exact cues were not elucidated ^18, 19^. Interestingly, other virulence factors such as InlJ or LLS are not expressed in cultured cells but are upregulated *in vivo* either in the liver and blood ^20^ or within the intestine ^21^, again upon undefined environmental cues. Thus, further work is required to determine what currently uncharacterized signals may be sensed by *Lm* for the upregulation and activation of virulence genes that are poorly expressed *in vitro*.

### Subversion of the host cytoskeleton

It has long been known that actin polymerization drives *Lm* host cell entry ^22^ as well as intracellular and intercellular motility ^23^. Once *Lm* reaches the host cytosol, ActA is transcriptionally upregulated and localized to the bacterial cell surface, where it recruits and activates the host actin regulator the actin-related protein 2/3 (Arp2/3) complex ^24^. The resulting actin cloud surrounding the bacteria enables it to evade detection by the host autophagy machinery ^25– 27^. ActA polarization at one of the bacterial cell poles results in a polarized polymerization of actin. The resulting actin “comet tails” propel the bacteria within the host cell cytosol and facilitate cell-to-cell spread. Although this process is well characterized, recent results have uncovered novel insights into the composition of the host Arp2/3 complex, how the actin cytoskeleton is involved in intracellular and intercellular motility, and how these processes are dependent on the stage of infection and subcellular context.

***Exploiting the Arp2/3 complex during infection.*** The Arp2/3 complex is composed of seven subunits: the Arp2 and Arp3 proteins and five Arp complex proteins (ARPC1–5) ^28– 32^. When activated by nucleation-promoting factors, it binds to a pre-existing actin filament and catalyzes the formation of a *de novo* Y-branched actin filament. Interestingly, *Lm* ActA mimics host nucleation-promoting factors to recruit and activate Arp2/3 near the bacterial surface ^32^.

We recently discovered differential requirements for subunits of the Arp2/3 complex for distinct aspects of *Lm* infection that require actin, i.e. entry and actin-based motility ^33^. Strikingly, ARPC1B, but not ARPC1A, appears to be critical for efficient *Lm* cell invasion. In contrast, ARPC1A, but not ARPC1B, is required for actin comet tail formation. Together, these results suggest that different isoforms of ARPC1 are exploited by *Lm* differently. Both ARPC4 and ARPC5 appear to be dispensable for cell invasion. In contrast, ARPC5 is not critical for actin tail formation. Thus, rather than existing as a single canonical complex, different Arp2/3 complexes may be formed by different subunits, and this modularity can be exploited by *Lm* for distinct steps of infection ^33^. The mechanism by which *Lm* can activate different Arp2/3 complexes and the effect of differential Arp2/3 activation on the actin cytoskeleton are still unknown.

It is currently unclear whether different Arp2/3 complexes exist and play a role *in vivo*. Nevertheless, the existence of different Arp2/3 complexes has also been recently reported in the case of focal adhesions ^34^ and the actin-driven intracellular propulsion of vaccinia virus ^35^. The recent discovery of sick but living human children with frameshift mutations in ARPC1B, the predominant ARPC1 isoform expressed in blood cells ^36^, suggests critical but distinct roles for different components of the Arp2/3 complex *in vivo*.

***Moving inside cells: mechanisms of *Lm* intracellular propulsion.*** Actin polymerization is known to propel intracellular *Lm,* but the precise mechanism of force generation has remained unclear. There are two prevailing models for actin polymerization-dependent intracellular propulsion of *Lm*. In the “Brownian ratchet” model, growing tangential actin filaments protrude and provide the propulsive force ^37^. The alternate “macroscale elastic propulsion” model implicates large-scale deformation of the actin meshwork as propelling the bacterium forward ^37^. Whether *Lm* intracellular propulsion is driven by individual actin filament elongation or by elasticity of the actin network was unclear.

Recent cryo-electron tomography of *Lm-*associated actin comet tails both within the cell ^38^ and within cell-free extracts ^39^ has shed some light on this process. The network of actin comet tails is composed of both branched and, surprisingly, some bundled filaments ^39^. The novel discoveries of additional F-actin bundles throughout the comet tail perpendicular to the direction of motion ^38^ in addition to tangentially orientated filaments to the bacterial surface suggest that elastic propulsion is the major driving force of *Lm* propulsion.

These studies are reminiscent of the debate concerning the lamellipodial actin network in migrating cells. The canonical view of Arp2/3-mediated branched actin networks of lamellipodia ^28– 31^ was challenged by a report implicating very little branched actin but instead many overlapping parallel actin bundles ^40^. The suggestion that actin filaments were mainly unbranched in lamellipodia was controversial ^41– 43^. Ultimately, a consensus was reached: lamellipodia are once again considered to contain Arp2/3-mediated branches of actin, but there are far fewer of them than expected ^44^. Membrane-tethered actin polymerizers are thought to mechanically and transiently link actin protrusions to the leading edge plasma membrane of a migratory cell ^37^. This transient F-actin polymerization model is similar to the model of actin propulsion of *Lm* ^37^. Altogether, these recent studies highlight the fruitful collaboration of studies of F-actin polymerization in cell migration and *Lm* propulsion.

In addition to actin comet tails, *Lm* also induces actin-based bacterial protrusions at the host cell plasma membrane to drive cell-to-cell spread. The actin network in *Lm*-mediated protrusions is composed of parallel actin filaments ^38^—more parallel and less branched than would be expected for Arp2/3-driven polymerization. The Rho-family GTPase cell division cycle protein 42 (Cdc42) is a conserved upstream regulator of host nucleation-promoting factors and Arp2/3 ^45, 46^ but has no role in *Lm* actin comet tail formation ^47^. In polarized epithelial tissue culture, *Lm* actin-based protrusions must counteract cortical tension. *Lm* partially relieves this tension by secreting the protein InlC, which inhibits Tuba, a Cdc42 activator ^48, 49^. While these results suggest that Cdc42 activity restricts *Lm* cell-to-cell spread, a subsequent report by another group suggests that membrane protrusion formation requires active Cdc42 and the actin regulator formin ^50^, which induce bundled F-actin. The reason for the conflicting requirements of Cdc42 activity for *Lm* cell-to-cell spread is unclear, although the authors speculate that the discrepancy may be because of the difference in cell types used (polarized epithelial Caco-2 versus non-polarized HeLa cells). Further work is required to ascertain the different requirements for Cdc42 activity in *Lm* intercellular spread and how the choice of model tissue culture affects these requirements.

Recently, new host cell factors that are recruited to the *Lm* comet tail were discovered. In addition to the known ActA targets Arp2/3 and enabled/vasodilator-stimulated phosphoprotein (Ena/VASP) ^51– 54^, ActA was recently shown to recruit lamellipodin. Lamellipodin is a binding partner of Ena/VASP and an actin regulator in lamellipodia ^55^ that promotes *Lm* cell-to-cell spread ^56^. Interestingly, lamellipodin is recruited to *Lm* actin comet tails independently of Ena/VASP, highlighting that lamellipodin can bind to F-actin. Although lamellipodin promotes cell-to-cell spread, curiously, lamellipodin knockdown increased the speed of actin-propelled *Lm* ^56^. How lamellipodin both promotes *Lm* intercellular spread and appears to reduce *Lm* comet tail speed remains to be clarified. Another group has found that lamellipodin can bind directly to F-actin independently of Ena/VASP *in vitro* and in cultured migratory cells, possibly promoting lamellipodial formation ^55^. Together, these results highlight the subversion of host cell lamellipodial formation by *Lm* to induce cell-to-cell spread.

***Actomyosin contractility and *Lm*.*** Non-muscle myosin II (myosin) is an actin-based motor protein that assembles into bipolar filaments to exert contractile forces. Interestingly, myosin is known to inhibit *Lm* infection ^48^. As mentioned above, suppression of Cdc42 activity in polarized epithelial cells favors cell-to-cell spread, presumably through relaxation of cortical tension ^48, 49^. However, direct quantification of the relaxation of cortical tension by *Lm* is lacking, and it would be interesting to measure tension as routinely performed in developmental biology research ^57, 58^. In addition, pharmacological inhibition of myosin was shown to favor *Lm* host cell adhesion and invasion ^59^. Phosphorylation of the myosin heavy chain at a conserved tyrosine residue was detected in response to *Lm* infection ^60^. Although phosphorylation of this tyrosine has been previously predicted in muscle myosin heavy chain ^61^, its impact on myosin contractility is unknown. Myosin activity seems to protect plasma membrane integrity from LLO-induced damage and this leads to increased host survival *in vivo* in a zebrafish infection model ^62^, although the underlying mechanism remains unclear.

Furthermore, formin and the actomyosin regulator Rho-associated kinase (ROCK) induce the internalization of *Lm* into endothelial cells ^63^. While in other cell types (such as epithelial and fibroblast) ROCK inhibits the entry of *Lm*, ROCK appears to favor bacterial adhesion to the cell surface of endothelia ^63^. It will be interesting to see if other regulators of actomyosin cortical tension cell–cell adhesions (for example 64) are involved in *Lm* infection.

### Subversion of host endoplasmic reticulum

*Lm* is known to alter the host endoplasmic reticulum (ER). Indeed, *Lm* induces ER stress and the unfolded protein response ^65^. The coat complex COPII, required for ER-to-Golgi trafficking ^66^, was recently found to restrict *Lm* cell-to-cell spread in polarized epithelial tissue culture ^67^. In addition, we discovered that *Lm* infection induces the expression of the small ubiquitin-like modifier interferon-stimulated gene 15 (ISG15) in non-phagocytic cells, triggering an ISGylation of a number of ER and Golgi proteins and increasing cytokine secretion ^68^. Furthermore, studies have uncovered a novel role for Gp96 (glycoprotein of 96kDa), an ER resident protein chaperone. *Lm* infection was already known to trigger Gp96 recruitment from the ER to the plasma membrane, becoming exposed to the cell surface and co-localizing with surface bacteria ^69, 70^. Recently, Gp96 was shown to be recruited to sites of LLO-induced blebbing along with myosin ^62^, but the underlying mechanisms are unclear. It will be interesting to see if there are other strategies used by *Lm* to perturb trafficking between endomembrane components, especially in the context of different cell types.

## *In vivo Listeria* behavior

### Overcoming physiological barriers

*Lm* pathogenesis relies on the ability of the bacterium to traverse several physiological barriers, including the intestinal epithelium and the placenta, and survive in multiple cell types ^71^.

Passage through the intestinal epithelial barrier is the first port of entry for *Lm* into the host. Interaction of the *Lm* surface protein InlA with E-Cadherin (E-cad), the host adherens junction epithelial cadherin is the key step in *Lm* intestinal invasion. Although E-Cad is localized to the basolateral membrane of vertebrate epithelial cells and would thus be generally inaccessible to *Lm* in the intestinal lumen, E-Cad is accessible at extruding cells at intestinal villi tips ^72^ and in mucus-secreting goblet cells ^73^. The interaction between InlA and human E-Cad is species specific ^74^. Thus, a knock-in transgenic mouse line bearing a point mutation in E-Cad that allows for the InlA–E-Cad interaction is used for oral infections with *Lm* ^75, 76^, although many studies are still performed with non-transgenic mice ^21^. InlA–E-Cad interaction triggers rapid transcytosis of *Lm* through goblet cells into the basal lamina propria ^73^. Recent work has shown that phosphoinositide 3-kinase (PI3-K) is constitutively active in the intestine, explaining why the *Lm* surface protein InlB, which is known to activate PI3-K, is not required for crossing the intestinal barrier ^77^. Interestingly, it was shown that *Lm* is mostly extracellular in the intestine of orally infected mice and that the intracellular pool is a minor but important fraction during infection ^78^. The majority of *Lm* in the gut was discovered to be associated with monocytes, but there is very poor intracellular growth ^79^ in these cells.

*Lm* is one of the few pathogens capable of traversing the placental barrier. It requires both InlA and InlB ^75, 80^. InlA-mediated invasion of *Lm* into the placenta requires InlB-dependent activation of PI3-K ^77^. Furthermore, a new Inl, InlP, has been discovered as an enhancer of placental invasion in both human placental explants and *in vivo* infection of guinea pigs and mice ^81^, although the mechanisms through which InlP acts remain to be elucidated.

## Interaction with the host gut microbiota

An emerging field of investigation is the interaction between the host gut microbiota and enteropathogens. Pre-colonization with lactobacilli protects mice against oral infection by *Lm* ^82^. Administration of *Lactobacillus* affects the expression of host genes and *Lm* protein-coding genes and small RNAs ^82^. In addition, the host gut microbiota interferes with the host microRNA (miRNA) response upon *Lm* oral infection ^83^. Recently, we uncovered that epidemic strains of *Lm* express a bacteriocin in the gut of orally infected mice, altering the host gut microbiota to favor *Lm* infection ^21^. It will be interesting to investigate whether *Lm* has other means with which to modulate the host gut microbiota. These results are beginning to uncover the interplay among the host, the host’s microbiota, and the enteropathogen *Lm.*

## Post-genomics era: considering more than just laboratory strains

The rise of fast genomic sequencing has opened new avenues to study *Lm*–host interactions. The plethora of genomic data and development of new bioinformatic tools have greatly facilitated the study and comparison of multiple *Lm* strains and other closely related *Listeria* species ^84, 85^. The development of proteogenomics and the integration of sequencing and mass spectrometry have uncovered novel anti-sense RNAs ^86^ and novel mini-proteins ^87^ of *Lm*. Unsurprisingly, different *Lm* strains possess differences at the genomic, transcriptomic, and pathogenic level ^85, 88^. For example, the novel *Lm* bacteriocin cited above that targets the host gut microbiota ^21^ is present in epidemic *Lm* strains but is absent in the standard reference laboratory strains. Certain epidemic strains appear more virulent in animal studies and are able to infect the central nervous system and traverse the placental barrier in human cases of listeriosis ^89^. In contrast, many of the reference laboratory strains are poorly neuroinvasive ^90^, suggesting that analysis of clinical isolates may be more fruitful for the investigation of human disease.

Recent genomic comparative studies of multiple strains, both laboratory and clinical ^84, 85, 90, 91^, including the recently sequenced 306 draft genomes of *Lm* isolates ^92^, have highlighted that analysis of *Listeria* biodiversity and genomic conservation is quite informative for the understanding of virulence. Identification of genomic regions over-represented in more virulent strains as well as differences at the transcriptomic level are promising ways to uncover novel bacterial factors involved in infection and in clinical hypervirulence. The recent development of Listeriomics, an online tool to easily compare sequenced *Listeria* species, should be very instrumental in this post-genomics approach ^93^.

## Conclusions and perspectives

Recent discoveries have advanced our understanding of *Listeria*–host interactions. Novel cues for the upregulation of virulence factors as well as the discovery of genes expressed exclusively *in vivo* highlight the need for consideration of the environment and tissues during *Lm* infection. In the near future, high-throughput sequencing and bioinformatics of multiple *Listeria* species will yield more insights into the mechanisms by which *Lm* subverts the host during infection *in vivo*.

## Abbreviations

ActA, actin assembly-inducing protein; Arp2/3, actin-related protein 2/3; ARPC(1–5), Arp complex proteins 1–5; Cdc42, cell division cycle protein 42; E-Cad, epithelial-cadherin; Ena/VASP, enabled/vasodilator-stimulated phosphoprotein; ER, endoplasmic reticulum; Gp96, glycoprotein of 96 kDa; Inl, internalin; ISG15, interferon-stimulated gene 15; LLO, listeriolysin O; *Lm*, *Listeria monocytogenes*; PI3-K, phosphoinositide 3-kinase; PrfA, positive regulatory factor A; ROCK, Rho-associated kinase.
